# Complete genome sequence of the *Niallia* sp. strain Kr1, a bacterium isolated from a geothermal power plant in Iceland

**DOI:** 10.1128/mra.00609-25

**Published:** 2025-09-24

**Authors:** Danae Bregnard, Diego Gonzalez, Deirdre E. Clark, Ásgerður K. Sigurðardóttir, Simona Regenspurg, Pilar Junier

**Affiliations:** 1 Laboratory of Microbiology, University of Neuchâtel 27214, Neuchâtel, Switzerland; 2 Geoenergy, GFZ German Research Centre for Geosciences, Potsdam, Germany; 3 ISOR, Iceland GeoSurvey, Kópavogur, Iceland; 4 Landsvirkjun, Reykjavík, Iceland; University of Southern California, Los Angeles, California, USA

**Keywords:** bacterial genome, extremophiles

## Abstract

We report the complete genome sequence of the *Niallia* sp. strain Kr1*,* a gram-negative, spore-forming bacterium isolated from the geothermal fluids of an Icelandic geothermal power plant injection well. This strain belongs to a putative new species within the *Niallia* genus.

## ANNOUNCEMENT

**Fig 1:**
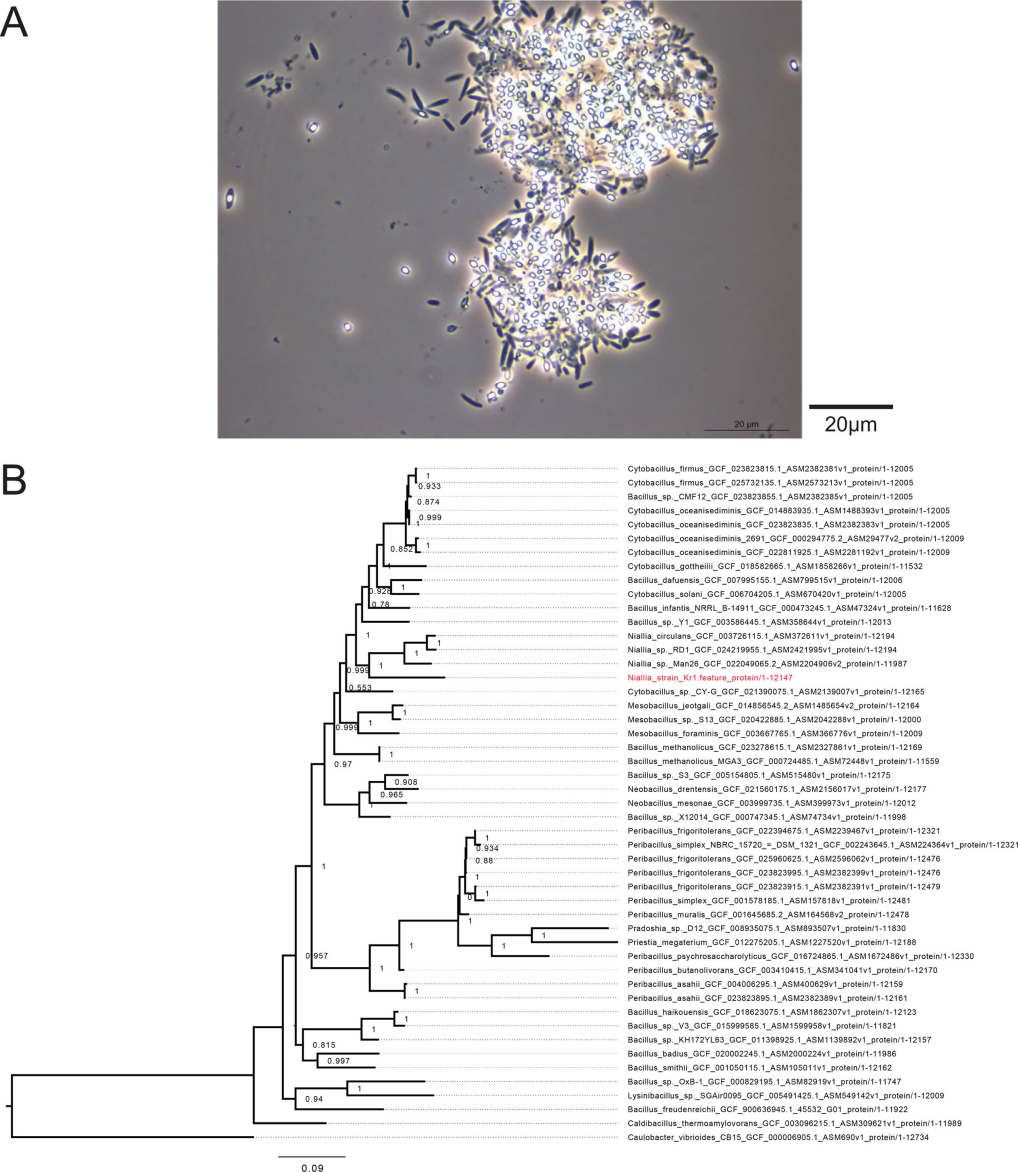
(**A**) Optical microscopy image of the strain Kr1 (vegetative cells and spores) under phase-contrast (spores appear bright) (5), using a Leica DMR Fluorescence microscope with a Leica DFC7000T camera . Scale bar represents 20 µm. (**B**) Phylogenetic tree based on 40 conserved proteins, placing the strain Kr1 within the *NNiallia* genus. The placement of strain Kr1 is highlighted in red.

The *Niallia* genus, recently separated from the *Bacillus* genus, encompasses eight bacterial species (1), including *Niallia circulans* (2–4), and *Niallia nealsonii*, isolated from the clean room of a space-craft assembly (5). Strain Kr1, a spore-forming bacterium (Fig. 1A), was isolated from geothermal fluids (well-head temperature: 106.8°C; 1L of fluids collected from the well-head in a sterile 1L Nalgen bottle; sample stored at 4°C until processing) collected from an electricity-producing power plant injection well in Iceland (65.7142293 N, 16.7303151 W). Enrichment was done in Marine Broth 2216 (BD Difco, USA) at 60°C without agitation (1 mL of sample for 25 mL of culture medium). Subsequent cultivation on solid Marine Broth 2216 (1.5% agar) led to the isolation of one bacterium.

Genomic DNA was extracted from a culture in Marine Broth 2216 (incubation at 30°C, 110 rpm for 48 h) using the Wizard HMW DNA Extraction Kit (Promega, USA). Sequencing was performed on a Pacific Biosciences Sequel II instrument after DNA shearing, size selection, and HiFi SMRTbell Library construction according to PacBio SMRTbell prep kit 3.0 recommendations (average insert size: 8,755 bp; chemistry v.11.1.0.154383). A full circular genome was assembled and circularized from 24,461 HiFi reads (default PacBio QC and trimming) using unicycler (6) (v.0.5.0), miniasm for assembly, and racon (v.1.5.0) for polishing (average coverage: 40 ×, N50: 8,658). The arbitrary start of the linearized sequence was positioned at the start of the *dnaA* gene using circlator (7) (v.1.5.5). One consistently methylated motif (Gm6AYNNNNNRTARC/GYTm6AYNNNNNRTC, >99% detection rate) was identified by Smrtlink (v.11). The genome was annotated using NCBI Prokaryotic Genome Annotation Pipeline (v.6.5). Assembly quality was assessed using CheckM (8) (estimated completeness 97.14%, contamination 6.25%).

The circular complete genome had 5,151,560 bps with a 37% G+C content, including 4,748 protein-coding genes, 17 copies of each ribosomal RNA gene (5S, 16S, 23S), 193 tRNAs, nine non-coding RNAs (signal recognition particle sRNA, 6S RNA, and RNA component of RNase P, and the transfer-messenger RNA); 82 additional putative protein-coding genes were predicted to be pseudogenes. The amino acid composition of the full proteome showed a limited ERK bias around −14 (9), which suggests that the strain is moderately thermophilic (predicted optimal growth temperature 50–60°C). Its presence in geothermal waters is likely due to the production of resistant spores, as all 78 genes considered essential for sporulation in Clostridia (10) were present in the genome. Endospores have been observed in other *Niallia* strains (5).

GTDB-tk (v. 2.20) (11) was not capable of assigning a rank beyond the order (Bacillales) to strain Kr1. Average nucleotide identity based on pyani (12) (v.0.2.12) gave the highest ANI values with *Peribacillus asahii* GCF_004006295.1 (73.93%) and with *N. circulans* GCF_003726095.1 (73.69%). A phylogenetic tree based on a set of ribosomal and translation-related proteins (elongation factor G/ L14/L15/L16/L17/L18/L2/L22/L23/L24/L3/L36/L4/L5/L6/S10/S11/S12/S13/S14/S17/S19/S3/S4/S5/S7/S8/Tu) from all complete Firmicute genomes available on NCBI by 1 June 2023, aligned with muscle (13) (v. 3.8.31) and analyzed with iqtree (14) (v. 3.0.0), placed strain Kr1 within the *Niallia* genus close to the *Cytobacillus* genus (Fig. 1B).

## Data Availability

This whole-genome shotgun project has been deposited at NCBI under the accession CP130945.1; SRAs are available under accession SRR27458440. This work was exempt from institutional ethics committee review. For all software, default parameters were used except where otherwise noted.
